# Identifying essential factors that influence user engagement with digital mental health tools in clinical care settings: Protocol for a Delphi study

**DOI:** 10.1177/20552076221129059

**Published:** 2022-10-11

**Authors:** Brian Lo, Quynh Pham, Sanjeev Sockalingam, David Wiljer, Gillian Strudwick

**Affiliations:** 1 Institute of Health Policy, Management and Evaluation, 7938 University of Toronto, Toronto, Ontario, Canada; 2 Campbell Family Mental Health Research Institute, 7978 Centre for Addiction and Mental Health, Toronto, Ontario, Canada; 3 Office of Education, 7978 Centre for Addiction and Mental Health, Toronto, Ontario, Canada; 4 Information Management Group, 7978 Centre for Addiction and Mental Health, Toronto, Ontario, Canada; 5 UHN Digital, 7989 University Health Network, Toronto, Ontario, Canada; 6 Centre for Digital Therapeutics, 7989 University Health Network, Toronto, Ontario, Canada; 7 Department of Psychiatry, Temerty Faculty of Medicine, 7938 University of Toronto, Toronto, Ontario, Canada

**Keywords:** User engagement, digital mental health, clinical care, Delphi, nursing informatics, psychiatry

## Abstract

**Introduction:**

Improving effective user engagement with digital mental health tools has become a priority in enabling the value of digital health. With increased interest from the mental health community in embedding digital health tools as part of care delivery, there is a need to examine and identify the essential factors in influencing user engagement with digital mental health tools in clinical care. The current study will use a Delphi approach to gain consensus from individuals with relevant experience and expertise (e.g. patients, clinicians and healthcare administrators) on factors that influence user engagement (i.e. an essential factor).

**Methods:**

Participants will be invited to complete up to four rounds of online surveys. The first round of the Delphi study comprises of reviewing existing factors identified in literature and commenting on whether any factors they believe are important are missing from the list. Subsequent rounds will involve asking participants to rate the perceived impact of each factor in influencing user engagement with digital mental health tools in clinical care contexts. This work is expected to consolidate the perspectives from relevant stakeholders and the academic literature to identify a core set of factors considered essential in influencing user engagement with digital mental health tools in clinical care contexts.

## Introduction

As the pandemic continues to impact the mental health of individuals,^1^ there is an urgent need to explore alternative approaches to supporting the overburdened mental health care system.^2^ The use of digital tools as part of clinical care, such as mobile apps, remote monitoring tools, patient portals and others, has been purported to support many of the emerging and ongoing challenges in mental health care, especially as they relate to addressing unique considerations brought about by the pandemic (e.g. social distancing).^3–5^ Moreover, with recent critiques highlighting the suboptimal impact of standalone digital mental health tools,^6^ there has been renewed interest from researchers and healthcare organizations to explore how these tools can be embedded in care delivery.^6–8^ For example, the World Health Organization,^9,10^ the American Psychiatric Association^11^ and the Mental Health Commission of Canada^12^ have all released campaigns and guidelines that advocate for and support the uptake of digital tools by clinicians as part of mental health care delivery. Clinical guidelines for anxiety and bipolar disorders, such as those from the Canadian Network for Mood and Anxiety Treatments, are also beginning to recommend the use of digital tools for relevant individuals.^13^

However, while there has been growing discussions and momentum on the uptake of digital mental health tools in clinical care, a number of emerging challenges (e.g. privacy and security) are becoming apparent that can jeopardize the use and value of digital health.^14,15^ Among the discussed challenges, there has been recognition of barriers that can lead to suboptimal and ineffective user engagement with the tool.^16–18^ In the current context, Perski et al. define user engagement as “(1) the extent (e.g. amount, frequency, duration and depth) of usage and (2) a subjective experience characterised by attention, interest and affect” (p. 258).^19^ Recent reviews on how end-users have engaged with mobile mental health apps have found that only a small fraction of users engage with the tool over time.^16,20^ However, in order to meaningfully enable its value, it is expected that effective engagement with the tool is necessary.^17,21–23^

### User engagement with digital health tools in clinical care

Since the increased recognition of the challenges related to suboptimal levels of effective engagement with digital health tools, there has been substantial interest in understanding ways to measure and support levels of effective engagement.^24^ For example, the Centre for Global eHealth Innovation at the University Health Network has developed an innovation to support real-time analysis and insights on how end-users engage with digital tools for chronic care.^25,26^ Other researchers in this field have also attempted to synthesize and identify the factors relevant to user engagement for digital tools.^21,22,27^ For example, in the work on mobile apps for trauma by Yeager et al., they proposed that user engagement may be influenced by the tool, the individual, and the environment.^28,29^ Building on this work, a number of studies, such as Cheung et al.*,* have also worked on developing interventions to support increased engagement with these tools.^30^

However, to our knowledge, there continues to be limited efforts on understanding the factors that impact user engagement that are specific to digital mental health tools that are used as part of clinical care delivery. A scoping review^31^ is being conducted to synthesize a list of factors that are relevant for user engagement with digital mental health tools in clinical care settings based on the components outlined in the Technology Acceptance Model^32,33^ and the Sociotechnical Model by Sittig and Singh.^34^ Given that the Sociotechnical Model^34^ encourages the investigation of relationships across the individual, organizational and system level, it is expected that considerations related to the end-users^35^ (e.g. previous experience), features of the tool^36^ (e.g. push notifications) and the clinical environment^37^(e.g. clinician buy-in) will be identified from this review.^31^ While these factors will help provide the foundation for understanding user engagement from research, it is expected that an overwhelming number of factors will be identified.^22^ As such, there is a need to develop a more practical framework with a succinct number of ‘core’ factors that can be used by digital health leaders to support the development and implementation of digital mental health tools in clinical care settings. Based on other studies in health informatics,^38,39^ there is an opportunity to collect the perspectives of relevant leaders and experts in this field in order to identify additional factors not included in the literature and to identify which of these factors strongly influence user engagement.^40^ Thus, this project aims to address these unmet needs through the use of consensus-gaining techniques.

### Study objective and aims

The objective of this study is to build on the findings from a scoping review conducted by the research team and identify a core set of factors that is considered by digital health experts to influence user engagement with digital mental health tools in clinical care settings by exploring the following research question: *What are the factors that are considered essential by digital health experts in influencing user engagement with digital mental health tools within clinical care contexts?* This objective will be explored through three aims:

Aim 1: Identify the perspectives of experts in digital mental health tools on the influence of the factors identified from the scoping review on user engagement with digital mental health tools in clinical care contexts.
Aim 2: Identify additional factors that are not discussed in the literature but may be considered essential for user engagement with digital mental health tools in clinical care contexts.
Aim 3: Establish consensus on the strength of each factor in influencing user engagement with digital mental health tools in clinical care contexts.

Through these three aims, it is expected that a core set of essential factors will be identified and validated based on the experience and knowledge from experts. Given that there is a growing diversity of digital mental health tools, the focus of this work is not to identify factors for a specific tool, but to provide the foundation for further adaptation and validation of these factors for different tools and clinical workflows.

## Methods

**Figure 1.**
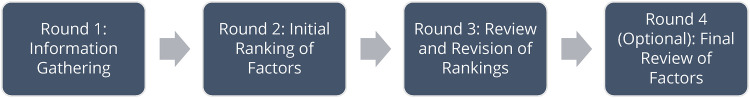
Overview of the Delphi technique.

In order to solicit the perspectives from individuals with relevant expertise in a systematic manner, a modified version of the Delphi approach will be used. The Delphi approach (Figure 1) is a consensus-gaining technique that allows for the collection of feedback from individuals in an asynchronous manner.^41–43^ In contrast to more traditional data collection approaches (e.g. focus groups), the Delphi approach uses multiple rounds of data collection in order to establish consensus among the participants of the panel.^42^ The first round is typically used to gather information from stakeholders about a topic. Subsequent rounds focus on using scales to examine consensus and similarity/differences in opinions across the expert panel. While its use can be traced back to the 1960s, it continues to gain popularity in use in health informatics on complex, emerging topics, such as digital compassion.^44,45^ The use of this technique has recently accelerated during the pandemic due to its advantage in collecting data asynchronously and in a virtual manner. According to Keeney and McKenna, there are various uses for the Delphi studies, including identifying consensus opinions on complex topics, exploring divergent and differing opinions among individuals and looking at differing policy options and alternatives.^46^

Given the relevance of this work for patients, clinicians and others, there is a need to ensure that patient and family perspectives are embedded in the development and delivery of this project.^47–50^ As such, in addition to including patients and caregivers as participants, a patient and family advisory representative will be consulted throughout the development, implementation, analysis and reporting of this study. Ethics approval has been obtained from the Research Ethics Board at the Centre for Addiction and Mental Health and the University of Toronto. 

### Participants and settings

There is considerable debate about who is considered an ‘expert’ or individual with sufficient expertise for the topic.^42^ While some roles have clear requirements by degree, professional certifications or job titles, significant variability remains in job titles and responsibilities across healthcare organizations.^51^ In the current context, there are many roles that can have relevance and expertise in supporting the engagement with digital mental health tools in clinical care settings. For example, patients and families are primary end-users of the tool and can bring lived experience in using this tool to support their care. Healthcare professionals are likely consulted in the use of these tools by their patients, and in some instances, play an active role in supporting the delivery of these tools. Other groups, including project managers, developers, healthcare administrators, may also have relevance in the development and implementation of digital mental health tools. Thus, as consistent with another Delphi study conducted in the United States,^52^ a diverse panel of individuals will be identified based on self-identified stakeholder group, self-reported experience and domain expertise.

In order to ensure that comprehensive insights are obtained on each of the factors related to user engagement, 20 to 40 individuals who identify as one of the following groups will be invited: (1) patients/caregivers, (2) clinicians, (3) healthcare administrators and policymakers, and (4) researchers. These groups are considered the main stakeholders relevant to the use of digital mental health tools in clinical care settings and an effort will be made to invite an equal amount of individuals for each group.^52^ While there is great variability in the number of individuals on a Delphi panel, this sample size is considered sufficient in previous studies in health informatics^39^ and appropriate for the time and resources available. Individuals who are eligible to participate in this study must self-report domain expertise and considerable experience in interacting with and/or utilizing digital mental health tools in clinical care settings over time. While there is currently no standard for quantifying experience and expertise in this area, several researchers within the research team have conducted the Delphi studies in health informatics. Building on the literature^52^ and their expertise, inclusion criteria as appropriate to each user group has been developed (Table 1). For example, healthcare administrators (e.g. product managers and directors) should self-report at least 3 years of experience supporting the development or implementation of a digital mental health tool being used in clinical care environments.

**Table 1.** List of inclusion criteria for each stakeholder group.

User group	Inclusion criteria
Healthcare administrators and policymakers	Self-report having at least 3 years of experience implementing and/or developing policy on the use of digital mental health tools in clinical care settings.
Healthcare professionals (e.g. physicians and nurses)	Self-report either: (1) at least 3 years of experience with using/supporting digital mental health tools as part of clinical care or (2) more than 50% of their care is delivered through and with digital mental health tools.
Patients and caregivers	Self-report that they are actively using a digital mental health tool as part of the mental health care they are receiving from a provider.
Researchers	Actively researching topics related to digital mental health tools that are used in clinical care mental health settings.

Based on the criteria above, a purposive sample will be obtained to ensure that there is sufficient diversity and expertise in this area among the expert panel. Recruitment of these individuals will be conducted using snowball sampling, as there is currently no central directory of individuals who meet the criteria for this study. A range of recruitment techniques will be used including distribution of recruitment materials through the professional network of the research team, social media, as well as through listservs of relevant digital health and mental health care organizations and working groups. Activities for recruitment are expected to begin in August 2022.

### Number of rounds

As mentioned earlier, data collection for the Delphi technique is conducted in multiple rounds until the threshold of consensus has been attained. However, there are no general guidelines on the number of rounds to complete for the Delphi study when consensus has not been reached for all statements. Keeney et al.^42^ suggested that developing a stopping rule a priori is beneficial as conducting too many rounds without clear benefits can be burdensome to the participants and detrimental to the overall success of the study. The sociotechnical framework by Sittig and Singh^34^ will be used to organize the factors included in the Delphi study and will be used to guide the project.

In this study, each round of feedback will be collected using an online REDCap survey. The survey will be sent to the email address of each participant and they are asked to complete it at a convenient time in a 2-week timeframe. A reminder will be provided after 1 week and each survey is expected to take 5 to 10 minutes to complete. The details for data collection and analysis for each round of surveys are consistent with methods used in the traditional Delphi studies^42^ and are described below.

### Round 1

#### Data collection

The main goal of the first round of the Delphi study is to collect demographics (e.g. gender, age, role and years of experience), assess each factor identified from the literature review and provide participants with the opportunity to review the content clarity of each factor. This is to allow participants to suggest any additional factors that they believe are important and should be included. In contrast to the traditional Delphi technique, modifications are made to the first round of the approach by seeding the list of factors from those who have been identified from the scoping review.^42^ This common modification allows the discussion to begin from the existing evidence base and reduces the burden on participants to exhaustively list all the factors that may be relevant. In this round, participants will be provided the name and a high-level description of each factor from the scoping review and asked to comment on the clarity of each factor. Examples of relevant factors can include technical requirements for the tool, information available on the platform and ease of use. For factors where clarity can be improved, participants are asked to explain how the name and/or description can be improved. In addition to the review of factors, individuals will be asked about their gender, age, role and years of experience. In order to understand the features of tools that will be examined in this study, participants will be asked to describe their previous experience with digital health tools and the tools they have used with their patients and families.

In order to identify any missing factors not surfaced in the literature, participants will be asked to comment on any factors that they believe are not captured in the list. Participants also have the option of requesting a phone call with the research team to further discuss factors not included in the list. A member of the research team will contact the participant to collect the missing factors for analysis.

#### Data analysis

Descriptive analysis will be used to analyze the demographic data and the perceived relevancy of each item collected in Round 1. For comments collected in this round, a content analysis^53^ will be used to identify the required revisions and additional factors that should be added to the existing list.

### Round 2

#### Data collection

In Round 2, the consensus-gaining process will begin. Using the revised list of factors, participants will be asked to rank the perceived strength of each factor on influencing user engagement with digital mental health tools in clinical care settings. There is significant variability in the type of Likert scales (e.g. number of points and balanced) and labels used for the ranking (e.g. of priority and of importance).^42^ Based on the objective of the Delphi study, various Likert scales are used to determine the level of consensus opinion among the expert panels. Given the objective of this work and the Likert scales used in previous health informatics Delphi studies,^39,52^ a 7-point Likert scale will be used from *Very Weak* to *Very Strong*.

#### Data analysis

Depending on the nature of the topic and objective of the Delphi, there are various approaches towards evaluating the ranking from the expert panel. Given that this work focuses on looking at the level of consensus, the median and interquartile ranges will be calculated to characterize the spread of responses for each factor in subsequent rounds. However, there is currently no gold standard definition for the threshold of defining consensus and what is considered an essential factor.^54^ In order to be consistent with the analysis approach for two related studies in health informatics, a factor is considered to have reached consensus if the interquartile range is ≤1.^39,52^ A factor is also considered essential if it has a median rating of 5 or higher on the 7-point Likert scale.

#### Rounds 3 and 4

In Round 3, participants will have the opportunity to review the rankings made by other participants and revise their ranking as necessary on factors that have not reached the consensus threshold. Through another online survey, participants will be presented with their own rating, as well as the median and interquartile range of the ratings from the panel for each factor. Based on this information, participants are asked to indicate if they would like to revise their rating, and if so, why. The explanation is used to contextualize the rationale for their change in ratings. A subsequent Round 4 is conducted if there are statements that still have not reached consensus using the threshold defined in data analysis for Round 2. The data analysis approach outlined in Round 2 will also be used here.

### Ethical considerations

Given the tight-knit informatics community in Canada, any identifying information from the comments will be removed in order to protect the identity of the participants throughout data collection. While the consenting process will be done remotely (due to the pandemic), the internationally accepted guidelines for the REDCap e-Consent process will be used.^55^ Participants can have as much time to review and discuss the study as they would like and will be reminded of the voluntary nature of being part of the study. For their time and efforts in the study, participants will be provided with a CAD$20 e-gift card.

## Discussion

To our knowledge, this will be one of the first studies that invite relevant experts to provide their perspectives on user engagement for digital mental health tools in clinical care contexts. Given the growing interest in improving effective user engagement and embedding digital health tools in clinical care settings, this study will provide ‘member checking’ of the findings from the scoping review, as well as uncover and capture the ‘tacit knowledge’ that exists in the digital health community about user engagement.^56^ The product from this study will be used to inform future efforts in evaluating and identifying opportunities for improving effective user engagement with existing digital mental health tools in clinical care contexts.

This approach aligns closely with the growing practice of engaging stakeholders in the development of guidelines and recommendations. The Delphi studies are becoming commonplace to support the involvement of service users and clinicians in developing clinical guidelines.^57^ In the last year, the use of consensus gaining techniques has grown to look at contemporary issues (e.g. artificial intelligence) in digital health^58^ as well as complex policy and practice issues.^52^ In the future, it may be useful to synthesize and examine the nuances and opportunities of leveraging consensus-gaining approaches in digital health research.

While this study will integrate findings from academic literature and that of experts in the field, several success factors and potential limitations should be kept in mind in the delivery of this work.^42,43^ Foremost, as the ratings and selection of factors are based on the perspectives of the panel of experts, selecting a diverse yet comprehensive panel of experts is essential for ensuring the validity of the findings. Moreover, over time, it is natural to expect that some participants will drop out of the study. While there is no rigorous guideline for the sample size of the Delphi studies,^42^ it would be useful to minimize participant drop out through reminders and personalized messages.
